# Vasculogenic and hematopoietic cellular progenitors are scattered within the prenatal mouse heart

**DOI:** 10.1007/s00418-014-1269-z

**Published:** 2014-09-09

**Authors:** Ewa Jankowska-Steifer, Maria Madej, Justyna Niderla-Bielińska, Sławomir Ruminski, Aleksandra Flaht-Zabost, Elzbieta Czarnowska, Grzegorz Gula, Dorota M. Radomska-Leśniewska, Anna Ratajska

**Affiliations:** 1 Department of Histology and Embryology, Medical University of Warsaw, Warsaw, Poland; 2 Student’s Scientific Group at the Department of Pathology, Medical University of Warsaw, Warsaw, Poland; 3 Department of Pathology, Center for Biostructure, Medical University of Warsaw, Chałubińskiego 5, 02-004 Warsaw, Poland; 4 Department of Pathology, Childrens’ Memorial Health Institute, Warsaw, Poland

**Keywords:** Vasculogenesis, Hematopoiesis, Embryonic mouse heart, Blood islands, Hemogenic endothelium

## Abstract

Vasculogenesis and hematopoiesis are co-localized in the embryonic body, but precise phenotypes of the cells contributing to these processes are not defined. The aim of this study was to characterize phenotypic profiles and location of putative vasculogenic and hematopoietic cellular progenitors in the embryonic mouse heart. Confocal microscopy, as well as ultrastructural and stereomicroscopic analyses, was performed on immunohistochemical whole-mount-stained or sectioned hearts at stages 11.5–14 dpc. A FASC analysis was conducted to quantify putative vasculogenic and hematopoietic cells. We found subepicardial blood islands in the form of foci of accumulation of cells belonging to erythroblastic and megakaryocytic lineages at various stages of maturation, exhibiting phenotypes: GATA2^+^/CD41^+^, GATA2^−^/CD41^+^, GATA2^+^/CD71^−^, GATA2^−^/CD71^+^, Fli1^+^/CD71^+^, Fli1^−^/CD71^+^, with a majority of cells expressing the Ter119 antigen, but none of them expressing Flk1. The subepicardium and the outflow tract endothelium were recognized to be the areas where progenitor cells were scattered or adjoining the endothelial cells. These progenitor cells were characterized as possessing the following antigens: CD45^+^/Fli1^+^, CD41^+^/Flk1^+^, Flk1^+^/Fli1^+^. A FACS analysis demonstrated that the CD41/Flk1 double-positive population of cells constituted 2.68 % of total cell population isolated from 12.5 dpc hearts. Vessels and tubules were positive for CD31, Flk1, Fli1, Tie2, including blood islands endothelia. The endocardial wall endothelia were found to function as an anchoring apparatus for megakaryocytes releasing platelets into the cardiac cavities. Phenotypic characteristics of vasculogenic (Flk1^+^/Fli1^+^) and hematopoietic (GATA2^+^/CD71^+^, CD41^+^/GATA2^+^) progenitors, as well as the putative hemogenic endothelium (Flk1^+^/CD41^+^) in embryonic mouse hearts, have been presented. Cardiac blood islands, the subepicardium and endothelium of the outflow tract cushions have been defined as areas where these progenitor cells can be found.

## Introduction

The cardiovascular system is the first one which develops and functions during the embryonic development. In mice, primitive circulation is established by embryonic day (ED) 8.5 (Drake and Fleming 2000). The cardiovascular system develops via vasculogenesis, i.e., de novo formation of blood vessels from a precursor cell—angioblast, and via angiogenesis, i.e., sprouting from the preexisting vessels. In the second case, precursor cells are endothelial cells of an existing vessel. It has been reported that hematopoiesis takes place during the prenatal formation of blood vessels and that vascular endothelial cells and hematopoietic cells are in a close relationship during ontogeny. Two main hypotheses propose an explanation of the close association of hematopoietic activity with the vascular lineage. The first postulates the existence of a common precursor of hematopoietic and endothelial lineages, the hemangioblast. The second one assumes the presence of hemogenic endothelium, which arises by dedifferentiation of mature endothelium into hematopoietic lineage. These two models describing the embryonic origin of hematopoietic cells have been regarded as mutually exclusive for many years. However, recent studies suggest that they may be combined and complement each other and that hemangioblasts may be linked to the hematopoietic cell progeny via a hemogenic endothelium (Lancrin et al. 2009; Antas et al. 2013).

Thus, the embryonic/fetal type of hematopoiesis has been described to originate from a hemogenic endothelium (Hirschi 2012). Hemogenic endothelial cells are rare cells found during embryonic development in the yolk sac, in the dorsal aorta at the level of the developing gonad/mesonephros, in the placenta, in the vitelline artery and in the umbilical artery (Antas et al. 2013), and very recently in the embryonic head (Li et al. 2012).

The heart itself possesses its own circulation that starts to develop at the postlooping stage, i.e., after 10 days postcoitus (dpc) (in mice), and becomes patent with the systemic circulation when the myocardial wall thickens. Coronary vessel formation proceeds also via vasculogenesis and via angiogenesis. Endothelial cell (EC) precursors and/or angioblasts invade the heart, differentiate into mature ECs, and form tubules and vessels.

The fetal heart has also been reported by several authors to be a place of hematopoiesis, with blood islands as particular spots of this biological event (Hirakow 1983; Ratajska et al. 2009; Red-Horse et al. 2010). These structures occur in mice at stages between 11 and 14 dpc. It has been recently demonstrated that apart from being present in blood islands, hemogenic activity exists in a subset of endocardial endothelial cells of the outflow tract cushions and atria of 8.25–8.5 dpc hearts (Nakano et al. 2013). These cells serve as a de novo source for transient definitive hematopoietic progenitors, earlier than in the aorta-gonad-mesonephros region. This hematopoietic activity would thus precede vasculogenic and angiogenic processes. In spite of these several reports, the data have not presented detailed characteristics of hematopoietic cells and their lineage affiliation. In addition, based on our own current observations, we cannot convincingly prove that hematopoiesis during later stages of mouse heart development (i.e., 11–14 dpc) is restricted entirely to cardiac blood islands. Studying the phenomena of neovascularization and hematopoiesis in normal mouse hearts in situ raises the problem of how to describe precisely the vasculogenic and hemogenic phenotypes of the endothelium.

There are several markers that would help to define these populations of ECs in situ. However, it must be pointed out that a single marker that makes it possible to distinguish hemogenic from nonhemogenic endothelial cells has not yet been identified. This fact impedes investigations on the embryonic origin and lineages of the hematopoietic cells.

Both endothelial and hematopoietic cells express a number of the same surface markers, such as CD31, CD34, Flk1, Tie2, c-kit, and transcription factors, such as Fli1 (Antas et al. 2013).

In intermediate stages of development between endothelial cell lining and free-floating blood cells that derive from this endothelium, up-regulation of c-kit, CD41 and CD45 has been documented (Boisset et al. 2010; Antas et al. 2013). Some hematopoiesis-specific membrane antigens, such as CD45, CD71, Ter119, and transcription factors Gata1, Gata2 are well characterized and demonstrated to be present in cells belonging to various hematopoietic lineages.

Due to close spatial and temporal relationships between the vasculogenesis and hematopoiesis and overlapping phenotypes of the endothelium involved in neovascularization and hematopoiesis, we propose to define the foci of these events by the use of multiple markers describing hematopoietic and vasculogenic activity in the developing heart in situ. Since previous reports indicated cardiac blood islands as spots of hematopoiesis, we were curious whether the rest of the heart area exhibits also similar activity. Thus, we performed our observations on the whole area of heart, including the subepicardial space, the myocardial wall and the endocardium, apart from the subepicardial blood islands. We focused on the time interval between 11.5 and 14 dpc, that is when blood islands are present and vasculogenic activity is most intensive in the developing heart.

Therefore, our aim was to locate within the mouse embryonic heart and characterize putative progenitor cells bearing hematopoietic and vasculogenic markers, using morphological and immunohistochemical methods. For this purpose, we used single, double or triple immunohistochemical labeling of the sets of surface markers and transcription factors. Our observations were performed on whole-mount-stained or serial-section-stained embryonic hearts. We used a confocal microscope analysis and an electron microscopy technique. A FACS analysis was performed to estimate the amount of the putative vasculogenic and hematopoietic cells in the embryonic heart.

## Materials and methods

### Animals

Experiments were performed on F1 cross of C57BL/6 and CBA mouse inbred strains. Second (II) Local Ethics Committee at the Medical University of Warsaw approved the experimental protocol for animal tissue harvesting. Experimental groups consisted of fetal mouse hearts taken from fetuses killed prenatally at 11.5, 12, 12.5, 12.75, 13.0, 13.5, 14.0 dpc. The hearts for individual experimental groups originated from different litters. Fetuses were dissected from cadaveric mothers (killed by overdose of xylamine/ketamine), hearts were excised and, depending on experimental method, either immediately fixed in Karnovsky’s fixative, or in 4 % buffered paraformaldehyde or frozen in liquid nitrogen after mounting in Tissue Freezing Medium (Leica Microsystems, Nussloch, Germany). For a FACS analysis, hearts from fetuses of 6 l (7–9 animals per litter) aged 12.5 dpc were excised.

### Confocal microscopy

Frozen sections were cut serially in a cryostat into 10-μm sections, fixed in an ice-cold acetone (at −20 °C) for 10 min, or in 4 % paraformaldehyde in PBS at room temperature for 20 min, and stained with a cocktail (double or triple combinations) of various species antibodies to different antigens for 1 h. The primary antibodies used: rat anti-CD68 and rat anti-CD41 (Abcam, Cambridge, UK); rabbit anti-Lyve1 (AngioBio Co. Del Mar, CA, USA); rabbit anti-Gata2 (Acris Antibodies GmbH, Herford, Germany); rat anti-CD45, rat anti-CD31, rat anti-CD71 and rat anti-Ter119 (BD Biosciences, Pharmingen, San Diego, CA, USA); goat anti-CD31 (Santa Cruz Biotechnology, Inc., USA); goat anti-neuropilin-1 (NP1, R&D Systems, Minneapolis, MN, USA), rabbit anti-Fli1 and rat anti-Tie2 (LifeSpan BioScienses, Inc, Seattle, WA, USA); and goat anti-Flk1 (R&D Systems, Minneapolis, MN, USA).

Subsequently, after three washes in PBS, the sections were incubated with donkey secondary antibodies to various species, i.e., conjugates: Cy3™ anti-rabbit IgG, Dylight™ 649 anti-rat IgG, Alexa Fluor 647 anti-rat IgG or FITC-anti-goat IgG (all secondary antibodies from Jackson ImmunoResearch Laboratories, West Grove, PA, USA) for 1 h, washed as above and counterstained with Hoechst (Sigma, St. Louis, MO) according to the manufacturer’s recipe.

Sections were mounted with DAKO immunofluorescent mounting medium (DAKO, Glostrup, Denmark) and viewed under a Leica confocal microscope, type TCS SP5.

### Whole-mount immunostaining

Paraformaldehyde-fixed hearts were washed 2 × 30 min in PBS at 4 °C, and subsequently 2 × 1 h in PBS-MT (PBS containing 0.1 % Triton X-100, 2 % skimmed milk) at room temperature. Incubation with blocking buffer, PBS containing 5 % BSA and 0.3 % TritonX-100 was carried overnight. On the following day, five subsequent washing steps in PBS-MT were performed: 2 × 1 h at 4 °C and 3 × 1 h at room temperature. Incubation with anti-Ter119 or anti-CD31 was carried overnight at 4 °C, and on the following day, the same washing steps were performed as before. Then, the primary antibody was bridged with polyclonal swine F(ab′)_2_ anti-rabbit-biotin (DAKO, Glostrup, Denmark), at dilution 1:500 in PBS-MT by overnight incubation as above, and washing steps as above. Next, the immunostaining was continued with peroxidase-conjugated streptavidin complex (Jackson ImmunoResearch Laboratories, West Grove, PA, USA) at dilution 1:500 in PBS-MT by overnight incubation at 4 °C. After washing steps in PBS-MT 2 × 1 h at 4 °C, and 3 × 1 h at room temperature, and finally in PBS-T (PBS + 0, 1 % Triton X-100 + 2 %BSA) for 2 × 20 min, the reaction was detected with peroxidase substrate diaminobenzidine tetrahydrochloride (DAB) (DAKO, Glostrup, Denmark), controlled under a microscope. The color reaction was stopped by washing the specimens 2 × 10 min in PBS, followed by fixation in 50 % methanol in PBS for 15 min and immersion in increased concentrations of glycerol: 50 % in PBS, 70 % in PBS and finally mounted in 100 % glycerol. Photography was performed using a Nikon stereomicroscope equipped with a digital system connected with a software program.

To improve imaging of the blood islands in mouse embryonic hearts, we used the method described by Mukouyama et al. (2012) with our own modification. In summary, 12.5 dpc hearts were isolated under the stereomicroscope and then the atria were removed carefully using micro-dissecting scissors. The hearts were divided into the ventral and dorsal surface with the use of a scalpel. After incubation with a mixture of antibodies, e.g., anti-Lyve1 (AngioBio, Del Mar, CA, USA), anti-CD31 (BD Biosciences, Pharmingen, San Diego, CA, USA) and anti-Flk1 (R&D Systems, Minneapolis, MN, USA) at 4 °C overnight, followed by washing steps, as described above, the specimens were incubated overnight with respective fluorochrome-conjugated secondary antibodies. After washing steps and Hoechst staining of nuclei, the specimens were mounted for a confocal microscope examination.

### Transmission electron microscopy

Hearts were fixed in ½ strength of Karnovsky’s fixative in 0.1 M cacodylate buffer pH 7.4, postfixed in 1 % osmium tetroxide and dehydrated in graded series of ethanol solutions and in propylene oxide. The material was embedded in Poly/Bed 812 (Polysciences, Inc. Warrington, PA, USA). Semithin sections stained with toluidine blue were analyzed under a light microscope. Selected fragments of hearts were cut for ultrathin sections. Specimens were double-stained with uranyl acetate and lead citrate and examined using Jeol 100 S (Jeol, Japan) transmission electron microscope.

### FACS analysis

12.5 dpc embryonic hearts were dissected out, rinsed with DMEM containing 1 % antibiotic/antimycotic solution and digested with Accutase (LifeTechnologies, Waltham, MA USA) at room temperature with agitation on a magnetic stirrer for 30 min. After a half of this time, cell suspension was carefully pipetted several times in order to disaggregate larger fragments of tissue. The dissociated cells were sieved through 40-µm mesh filter, washed with PBS twice, counted and stained for a flow cytometry analysis. Blood samples were collected by bleeding dissected fetuses in 5 ml of DMEM containing a 1 % antibiotic/antimycotic solution and before staining washed twice with PBS and counted. Both the cells isolated from the hearts and the blood cells from fetuses of whole litter were washed once with FACS buffer (PBS with 1 % bovine serum albumin and 0.09 % sodium azide). Afterward, cells were re-suspended in 100 µl of FACS buffer. For surface marker detection, 10 µl of both CD41-PE and Flk1-APC rat monoclonal antibodies (BD Biosciences, Pharmingen, San Diego, CA, USA) were added to each sample for a 30-min incubation at room temperature. Subsequently, the cells were washed once with FACS buffer and re-suspended in fresh buffer with 1 % paraformaldehyde. Samples were stored at 4 °C, and the analysis was performed within 72 h of staining using the FACSCalibur cytometer (BD Biosciences, Pharmingen, San Diego, CA, USA). Immediately before the analysis, MultiComp Beads (BD Biosciences, Pharmingen, San Diego, CA, USA) were stained with the antibodies and the beads were analyzed to compensate for spectral overlap between the PE fluorochrome and the APC channel. For each sample, 1 × 10^5^ events were acquired. Unstained samples were acquired as a reference. Data were analyzed with FlowJo software (Tree Star Inc.). The analysis gate was adjusted to exclude cell debris and erythrocytes with very low forward and side scatter values. Fluorescence signals of stained samples were compared with unstained controls to select a double-positive cell population. The percentages of double-positive cells for heart and blood samples of six litters (*n* = 6) were analyzed using a STATISTICA software package with the nonparametric Mann–Whitney *U* test to asses statistical significance. The *p* value of <0.01 was considered to be statistically significant.

## Results

### Blood islands are located subepicardially in both interventricular sulci and consist of endothelial and hematopoietic cells

Based on a spatial configuration of the endothelial cell marker—CD31 and the erythroblastic marker—Ter119, we can demonstrate blood island locations in the embryonic hearts at stages 11.5, 12, 12.5, 12.75, 13, 13.5, 14 dpc.

**Fig. 1:**
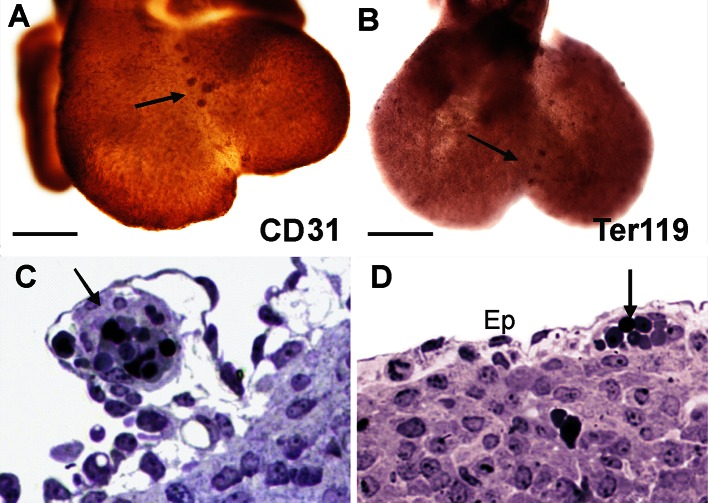
Subepicardial blood islands are positioned within interventricular sulci. **a**, **b** Whole-mount-stained 12 dpc hearts; anti-CD31 specificity—(**a**) and anti-Ter119 specificity—(**b**) demonstrate locations of blood islands in interventricular sulci; **c**, **d** semithin sections of a 13 dpc heart stained with toluidine *blue*. Blood islands (*marked with black arrows*) are positioned below the epicardium (Ep). The shape of blood islands is mostly spherical. *Scale bars* 50 µm

The first cardiac blood islands were found at 11.5 dpc stage, and they were localized only on the dorsal surface of the heart. In later stages (from 12.0 to 12.75 dpc), their number increased; a quantitative analysis (Table 1) indicated that the number of blood islands was higher on the ventral surface as compared with that of the dorsal surface of the heart. Blood islands were positioned distally from the stream of blood that washes the endocardium. They were found in the subepicardial mesenchyme of dorsal and ventral interventricular sulcuses and close to apex incisure of the heart (Fig. 1a–d).

**Table 1:** The number of blood islands in selected hearts of 11.5–14 dpc fetuses whole-mount immunostained with anti-Ter119 or anti-CD31 antibodies

dpc	Heart #1		Heart #2		Heart #3		Heart #4		Heart #5	
	Dorsal	Ventral	Dorsal	Ventral	Dorsal	Ventral	Dorsal	Ventral	Dorsal	Ventral
11.5	0	0	1	0	4	0	0	0	0	0
12	2	1	0	2	0	7	0	6	0	3
12.5	3	4	0	4	0	7	0	6	1	3
12.75	0	2	1	0	1	2	0	3	0	9
13	1	0	0	0	0	3	0	5	1	3
13.5	0	3	0	8	0	7	0	1	0	4
14	0	6	0	1	0	3	0	1	0	3

In every stage group, blood islands were counted on dorsal and ventral surfaces of five hearts

In 13.5 and 14 dpc hearts, blood islands disappeared on the dorsal surface, although there were several of them on the ventral surface of the heart.

**Fig. 2:**
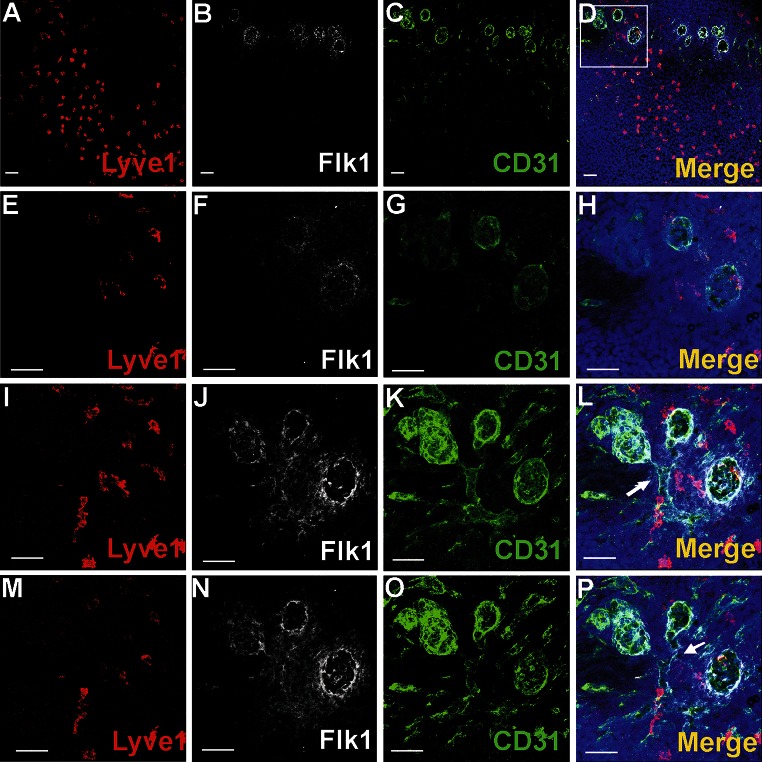
Blood island integration with forming coronary vessels. **a**–**p** represent a whole-mount-stained 12.5 dpc heart with the following combination of antibodies: anti-Lyve1 (*red*) (**a**, **e**, **i**, **m**), anti-Flk1 (*white*) (**b**, **f**, **j**, **n**) and anti-CD31 (*green*) (**c**, **g**, **k**, **o**). Blood islands are located in the ventral interventricular sulcus (**a**–**d**). The merged panel at a low magnification (**d**) and the area boxed in **d** are presented at different scanning levels at a high magnification in (**h**, **l**, **p**) and are counterstained in *blue* with Hoechst to visualize cell nuclei. Protrusions of blood islands coalescing with blood vessels are indicated with white arrows (**l**, **p**). *Scale bars* 50 µm

At spots of active angiogenesis, the blood islands began to change their shape from spherical to tubular. At stage 12 dpc and later, some of the blood islands gave protrusions directed toward the myocardium. Some of those protrusions then branched and coalesced, forming tubules, that finally fused with just-forming coronary vessels. This occurred particularly on the dorsal surface of the heart at stages 12–13 dpc, as confirmed by immunohistochemical observations of whole-mount-stained 12.5 dpc hearts (Fig. 2).

**Fig. 3:**
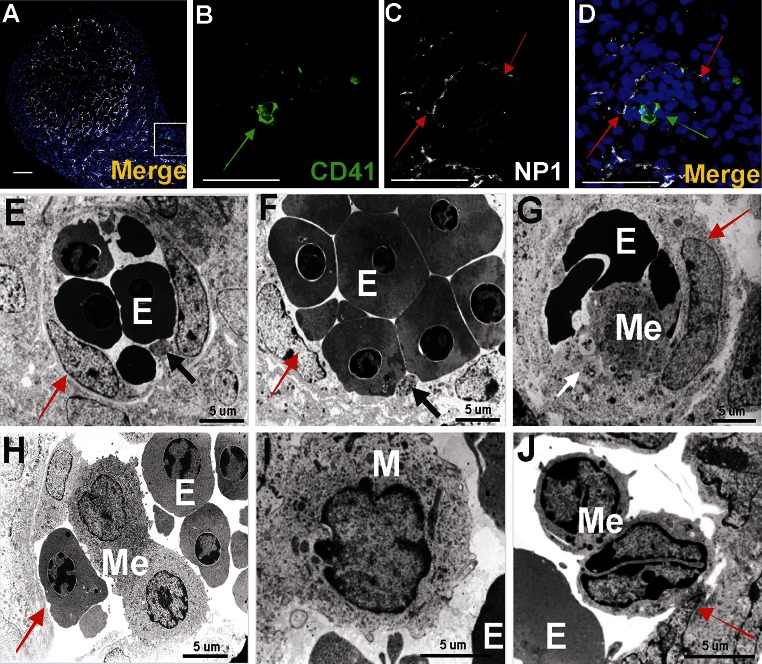
Different cell types are constituents of the subepicardial blood islands (**a**–**j**). **a**, **b**, **c**, **d** Confocal microscopy images of sections from a 13 dpc heart stained with anti-CD41 (*green*) (**b**), anti-NP1 (*white*) (**c**) antibodies and the merged picture stained additionally with Hoechst (*blue*) (**a**, **d**). The blood island shown in the *white box* of panel **a** is enlarged on panels **b**–**d**. Endothelial cells of blood island express NP1 (*red arrows*) (**c**, **d**), whereas CD41^+^ cells (*green*) (**b**, **d**) are present inside of the blood island. *Scale bars* 50 µm. **e**, **f**, **g**, **h**, **i**, **j** Electron microscopic pictures of blood islands. Cells with the ultrastructural features of endothelium positioned at the periphery of blood islands are indicated by *red arrows*. The interior of blood islands is represented by the following hematopoietic lineages: erythroblastic (E) (**e**–**j**) and megakaryoblastic (Me) (**g**, **h**, **j**); proplatelets occur adjacent to some Me (*white arrow* in **g**,) and other Me are devoid of proplatelets (**j**). Platelets (*black arrows*) (**e**, **f**) are numerous components of blood islands, whereas mesenchymal-like cells (M) are rarely found (**i**)

Cells at the periphery of blood islands expressed the blood vessel endothelial markers: CD31^+^/NP1^+^/Flk1^+^/Fli1^+^ (Figs. 2b, c, f, g, j, k, n, o, 3c, d; for Fli1—data not shown). They were negative for Lyve1, CD41 and Gata2. These endothelial cells were usually elongated, with the cytoplasm lightly stained, rich in polyribosomes, moderately developed rough endoplasmic reticulum (RER), and a few small electron-dense mitochondria (Fig. 3e–g). Their nuclei were rich in euchromatin, occasionally contained prominent nucleoli and exhibited deep infoldings.

### The cells present within blood islands belong to erythroblastic and megakaryocytic lineages

The predominant cell type found inside the blood islands belonged to the erythroblastic lineage. These cells expressed the Ter119 and CD71 antigens (Figs. 1b, 5b, g, e, j).

**Fig. 4:**
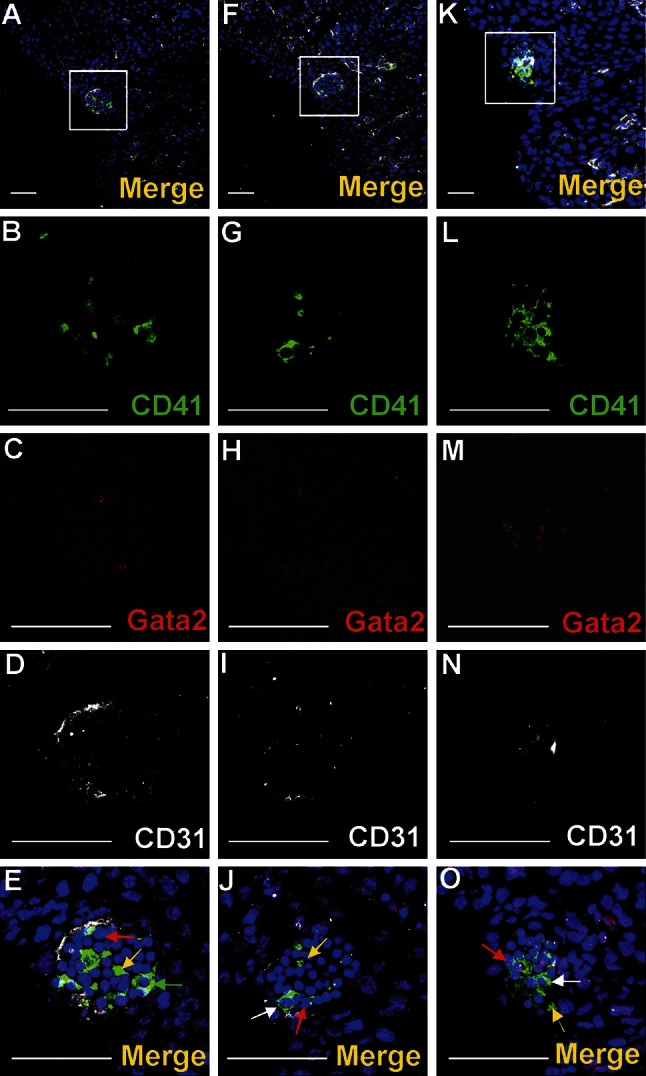
Diversity of CD41 and Gata2 expression in blood islands. Confocal microscope images of sections from a 13 dpc heart stained with anti-CD41 (*green*) (**b**, **g**, **l**), anti-Gata2 (*red*) (**c**, **h**, **m**), anti-CD31 (*white*) (**d**, **i**, **n**) antibodies; merged panels showing nuclei counterstained with Hoechst (*blue*) (**a**, **f**, **k**, **e**, **j**, **o**). Merged images on **e**, **j**, **o** demonstrate enlargements of areas *boxed* in **a**, **f**, **k**. Panels arranged in *horizontal lines* (i.e., **a**, **f**, **k**) represent selected Z-stacks of the same blood island. A diversity of CD41 and Gata2 expression is observed inside blood islands. Some cells co-express both markers (*white arrows*) (**j**, **o**). However, cells with only one antigen, CD41 (*green arrow*) (**e**) or Gata2 (*red arrows*) (**e**, **j**, **o**) are also present. The abundant population consists of cells *negative* for both markers. Small fragments of cytoplasm, presumably platelets, are *positive* for the CD41 marker (*yellow arrows*) (**e**, **j**, **o)**. *Scale bars* 50 µm

Nucleated cells expressing CD41 as well as small cytoplasmic fragments devoid of the nucleus (presumably blood proplatelets/platelets) were also detected within blood islands (Figs. 3a, b, d, 4a, b, e, f, g, j, k, l, o). CD41 expression was found in cells with a round nuclei and a thin rim of cytoplasm as well as in cells with abundant cytoplasm. The CD41 is specific for progenitor cells of hematopoietic lineage and for megakaryocytes (Li et al. 2005). Cells containing the CD41 antigen did not express Flk1, CD31 or transcription factor Fli1.

#### Blood islands did not contain Lyve1-positive cells

**Fig. 5:**
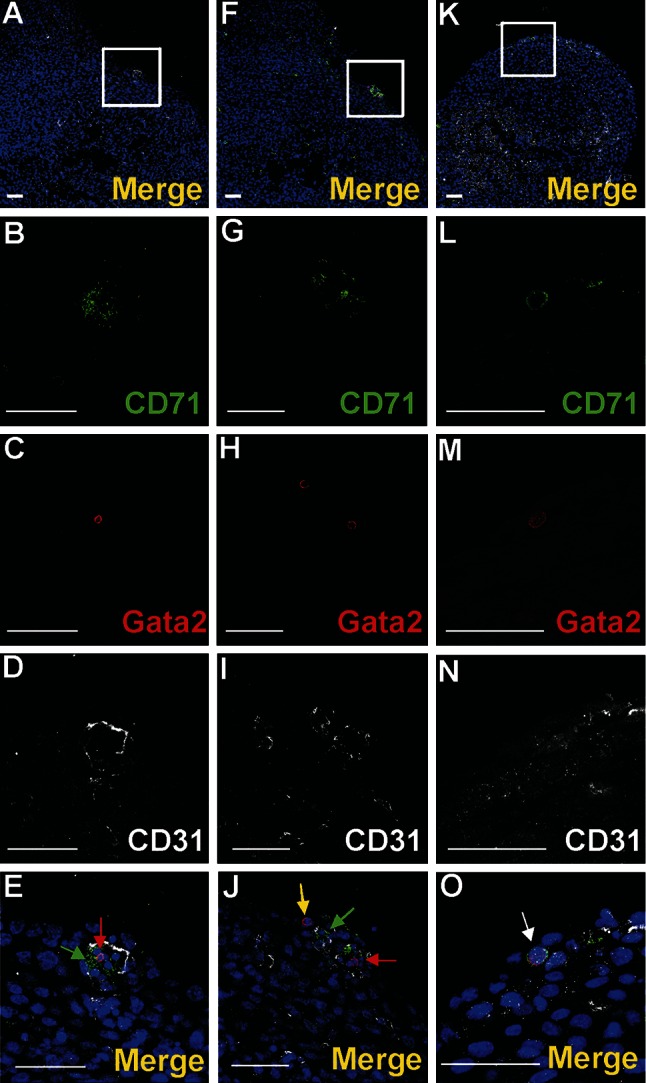
Subepicardial expression of CD71 and Gata2 markers. Confocal microscope images of sections from 13 dpc hearts stained with anti-CD71 (*green*) (**b**, **g**, **l**), anti-Gata2 (*red*) (**c**, **h**, **m**), anti-CD31 (*white*) (**d**, **i**, **n**) antibodies; merged panels showing nuclei counterstained with Hoechst (*blue*) (**a**, **f**, **k**, **e**, **j**, **o**). The subepicardial areas shown in *white boxes* of panels **a**, **f**, **k** are enlarged on panels **e**, **j**, **o**. Central part of blood islands consists of various cell populations: CD71^+^/Gata2^−^ (*green arrows*) (**e**, **j**), CD71^−^/Gata2^+^ (*red arrows*) (**e**, **j**) as well as cells *negative* for both markers. A few CD71^−^/Gata2^+^ cells (*yellow arrow*) are present in the subepicardium, outside of blood islands (**j**). Double-positive cells (CD71/Gata2) (*white arrow* in panel **o**) are located in close proximity to endothelial cells (CD31-positive). *Scale bars* 50 µm

Transcription factors Gata2 or Fli1 were expressed in a low number of cells. Cells exhibiting various phenotypes, such as Gata2^+^/CD41^+^, Gata2^−^/CD41^+^ (Fig. 4), Gata2^+^/CD71^−^, Gata2^−^/CD71^+^ (Fig. 5a–j), Fli1^+^/CD71^+^ and Fli1^−^/CD71^+^ (data not shown), were observed within the lumen of blood islands.

Electron microscopic observations confirmed that erythroblasts at different stages of differentiation were present inside blood islands. The cytoplasm of erythroblasts was of various electron density, reflecting the steps of their differentiation. At least three degrees of erythroblast cytoplasmic electron density were observed (Fig. 3e–h).

Most erythroblasts located in blood islands were in late stages of development, similar to these found in the circulation. Only a few erythroblasts in blood islands exhibited features of very early developmental stages.

**Fig. 6:**
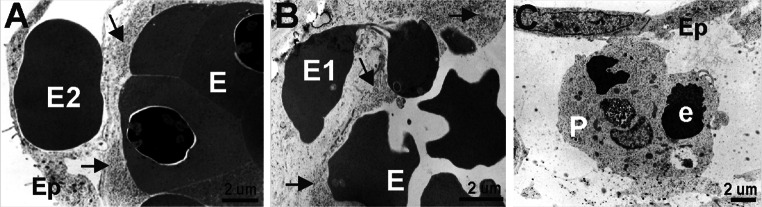
Erythroblasts are located in different areas of the subepicardium. Electron microscopic images demonstrating the location of erythroblasts inside blood islands (E) (**a**, **b**) and erythrocytes outside blood islands (E2) (**a**). Migratory activity of an erythroblast (E1) from a blood island into the subepicardial space is depicted in panel **b**. A phagocytozing cell (P) that contains remnants of erythroblast (e) cytoplasm and/or nuclei is present in the subepicardium (**c**). *Black arrows* point to endothelial cells of blood islands, and Ep indicates the epicardial lining

Occasionally, erythroblasts forming cellular processes toward endothelial cells were observed; presumptively, these processes were signs of migration of erythroblasts outside of blood islands or outside of young vessels (Fig. 6b). We can presume that these migrating cells are a source of freely ‘floating’ erythroblasts found in the subepicardial area (Fig. 6a). Signs of erythroblasts phagocytosis by the subepicardially located cells were detected (Fig. 6c).

Megakaryocytes and platelets were the second common component of blood islands (Fig. 3e–h, j). Megakaryocytes were characterized by diverse morphology. Some of them were large, with abundant electron-light cytoplasm rich in granules and narrow cisterns of endoplasmic reticulum. Their nuclei had irregular outlines (Fig. 3h). Other megakaryocytes were smaller and poor in cytoplasm with a high nuclear/cytoplasmic ratio. The nuclei of such megakaryocytes were deeply lobulated, with substantial amount of heterochromatin associated mostly with the nuclear envelope (Fig. 3j). In most blood islands, platelets filled the spaces between densely packed erythroblasts (Fig. 3e–g). Proplatelets (and/or platelets) sporadically formed protrusions contacting with the surface of endothelial cells. In a few cases, cytoplasmic projections penetrated between endothelial cells into the surrounding mesenchyme.

Occasionally undifferentiated mesenchymal-like cells were visible in blood islands (Fig. 3i). They were oval, contained light cytoplasm with a moderate number of organelles (mitochondria, free polyribosomes, cisterns of RER) and large nuclei, rich in euchromatin, with heterochromatin clumps located peripherally.

### Single cells residing in the subepicardium exhibit phenotypes of progenitor cells

**Fig. 7:**
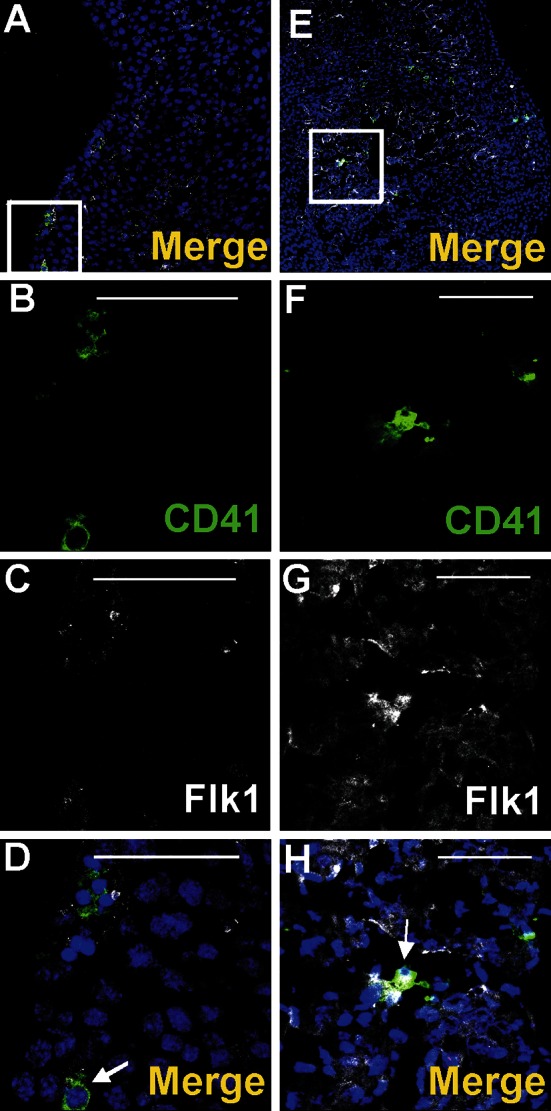
Cells co-expressing an endothelial cell/hemangioblastic marker (Flk1) and a hematopoietic marker (CD41) are located in the subepicardial space (**a**–**d**) and in the heart ventricles (**e**–**h**). Confocal microscope images of sections from 13 dpc hearts stained with anti-CD41 (*green*) (**b**, **f**), anti-Flk1 (*white*) (**c**, **g**) antibodies; merged panels include Hoechst staining of nuclei (*blue*) (**a**, **d**, **e**, **h**). The areas located in *white boxes* of panels **a**, **e** are enlarged on panels **d**, **h**. CD41/Flk1 double-positive cells (*white arrows*) are located in the subepicardial space (**d**) and as adhering to the ventricular endocardial endothelium (**h**). *Scale bars* 50 µm

**Fig. 8:**
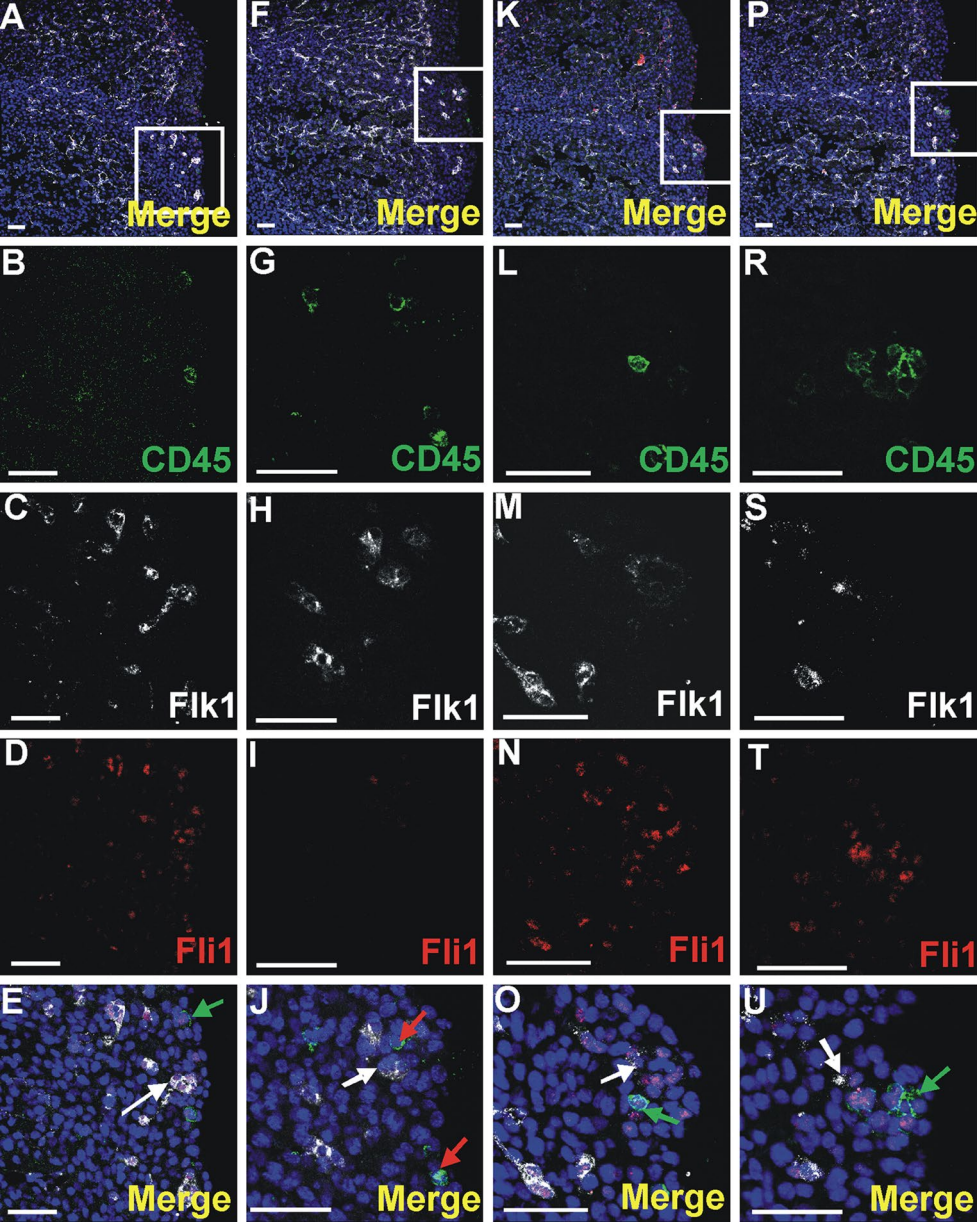
Cells expressing CD45 are scattered in the subepicardium of the interventricular sulci. Confocal microscopic images of sections from a 13 dpc heart stained with anti-CD45 (*green*) (**b**, **g**, **l**, **r**), anti-Flk1 (*white*) (**c**, **h**, **m**, **s**), anti-Fli1 (*red*) (**d**, **i**, **n**, **t)** antibodies; merged panels stained additionally with the Hoechst to depict cell nuclei (*blue*) (**a**, **f**, **k**, **p**, **e**, **j**, **o**, **u**). The subepicardial areas shown in *white boxes* of panels **a**, **f**, **k**, **p** are enlarged on panels **e**, **j**, **o**, **u**. Cells expressing CD45 phenotype are located outside blood islands in the subepicardium (*red arrows*) (**j**). Some of the CD45^+^ cells co-express the Fli1 transcription factor (marked with *green arrows*) (**e**, **o**, **u**). CD45^+^ and CD45/Fli1 double-positive cells are located in a close vicinity of Flk1^+^/Fli1^+^ cells (*white arrows*) (**e**, **j**, **o**, **u**). *Scale bars* 50 µm

**Fig. 9:**
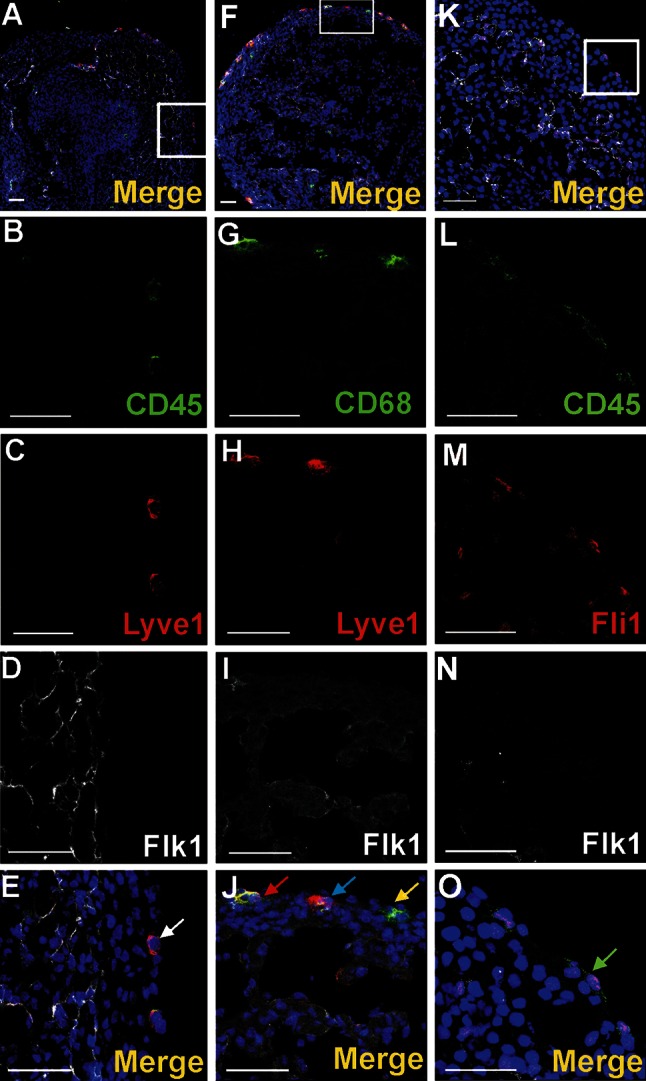
Cells of subepicardium express a diverse composition of markers. Sections from 13 dpc hearts stained with anti-CD45 (**b**, **l**) (*green*), anti-CD68 (**g**), anti-Lyve1 (**c**, **h**) or anti-Fli1 (**m**) (*red*), anti-Flk1 (**d**, **l**, **n**) (*white*) antibodies; merged panels that include Hoechst staining of nuclei (*blue*) are visible in **a**, **f**, **k**, **e**, **j**, **o**. The areas located in *white boxes* of panels **a**, **f**, **k** are enlarged on panels **e**, **j**, **o**. Subepicardially located cells bearing Lyve1 antigen can co-express CD45 antigen (*white arrow* in **e**) or CD68 (*blue arrow* in **j**) or can be Lyve1/CD68/Flk1 triple-positive (*red arrow* in **j**). There are also cells bearing CD68 but not Lyve1 and Flk1 (*yellow arrow* in **j**). Some cells of the subepicardium are CD45/Fli1 double-positive and do not express Flk1 (*green arrow* in **o**). *Scale bars* 50 µm

Cells expressing CD41/Flk1 markers were found occasionally in the subepicardial area (Fig. 7a–d). They were located outside of blood islands in scattered positions. CD41-positive cells were rarely located in the vicinity of the blood islands (Fig. 3a–d). Cells expressing CD45 were more numerous as compared to those CD41^+^ and were found mainly in the subepicardium of interventricular sulci (Fig. 8a, f, k, p); however, they were also present in other regions of the subepicardium (Fig. 9a–e, k–o). Some cells exhibiting the CD45^+^/Fli1^+^ phenotype and CD45-positive cells were scattered or located in the vicinity of Flk1^+^/Fli1^+^ cells (Fig. 8b–e, g–j, r–u). Occasionally, CD45^+^/Fli1^+^ cells were seen adjacent to CD45^−^/Fli1^+^ cells (Fig. 9k–o). Cells expressing the CD45^+^/Lyve1^+^ phenotype were also present in the subepicardium (Fig. 9a–e). Cells expressing CD45 did not possess NP1, Flk1 or Gata2 antigens.

CD68 marker expression was found in scattered subepicardial cells, and some of them co-expressed the Lyve1 antigen (Fig. 9f–j). Although Lyve1-positive cells presented an abundant population of the subepicardium, these cells were never observed in the lumen of blood islands.

**Fig. 10:**
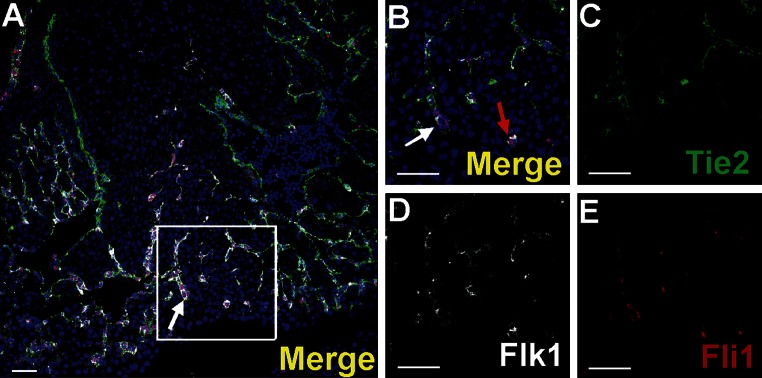
Heterogeneity of markers expressed in endothelial cells of the dorsal interventricular sulcus. Confocal microscope images of a 12.0 dpc heart sectioned transversely at the level of ventricles. Staining with anti-Tie2 (*green*) (**c**), anti-Flk1 (*white*) (**d**), anti-Fli1 (*red*) (**e**) antibodies. The picture **a** presents a merged panel of the heart area in a small magnification; the boxed region is demonstrated at a high magnification in **b**–e. Most cells with the endothelial markers Flk1 and Fli1 are also *positive* for Tie2 (*white arrows*) (**a**, **b**); however, some Flk1^+^/Fli1^+^ cells do not express the Tie2 antigen (*red arrow*) (**b**). *Scale bars* 50 µm

**Fig. 11:**
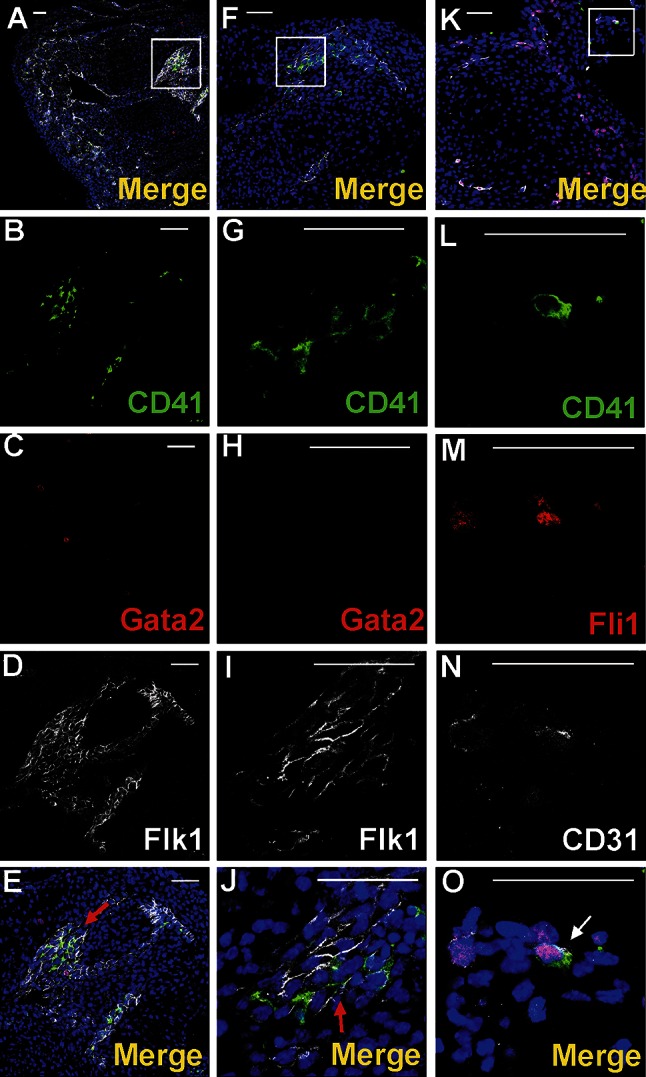
A subpopulation of endocardial endothelial cells expresses certain hematopoietic and vasculogenic markers. 12 dpc hearts sectioned transversely at the level of the dorsal atrioventricular sulcus stained with anti-CD41 (*green*) (**b**, **g**, **l**), anti-Gata2 (*red*) (**c**, **h)**, anti-Fli1 (*red*) (**m**), anti-Flk1 (**d**, **i**) and anti-CD31 (**n**) (*white*) antibodies; merged panels include Hoechst staining of nuclei (*blue*) (**a**, **f**, **k**, **e**, **j**, **o**). Areas marked with *white boxes* (**a**, **f**, **k**) are enlarged in **e**, **j**, **o**. CD41/Flk1 expressing cells in endothelial lining of endocardial cushions are marked with *red arrows* (**e**, **j**). A CD41/CD31/Fli1 triple-positive cell is located in atrial endothelial lining (marked with the *white arrow* in **o**). *Scale bars* 50 µm

A small number of Gata2-positive cells were found in the subepicardium and in the close location to blood islands. This transcription factor exhibited a strong expression. Generally, Gata2-positive cells did not bear the CD71 antigen; however, one cell expressing the CD71^+^/Gata2^+^ phenotype was found in the subepicardium (Fig. 5k–o) indicating that these cells were rare. On the contrary, Fli1 was strongly expressed in numerous cells located in the subepicardium, especially in the region of the atrio-ventricular sulcus (Fig. 10). Fli1-positive cells predominantly co-expressed also CD31, Tie2, and Flk1 molecules (Figs. 10, 11m–o); however, Fli1^+^/CD31^−^ cells were also present in the subepicardium (data not shown).

Endothelial cells forming coronary vessels co-expressed Flk1, Fli1 and Tie2 markers. However, in the subepicardial area, a few scattered cells bearing Flk1 and Fli1 antigens were negative for the Tie2 marker (Fig. 10).

Electron microscopic investigations indicated the presence of scattered cells of different ultrastructure in this region. Oval or elongated cells with the light cytoplasm containing few organelles were most abundant. They contained large nuclei, rich in euchromatin, whereas heterochromatin was located peripherally or formed irregular clumps inside the cell nucleus.

Another cell type, of more diversified shapes, was characterized by the presence of rich RER and prevailed euchromatin in the nuclei. In the subepicardium, numerous large phagocytozing cells were also present (Fig. 6c). These cells usually contained irregular nuclei and electron-dense, prominent fragments of the phagocytozed cytoplasm and/or a nucleus of erythroblasts and small dark mitochondria and moderately developed RER cisterns.

### A subpopulation of endocardial endothelium exhibits phenotypes of putative hematopoietic and vasculogenic progenitors

**Fig. 12:**
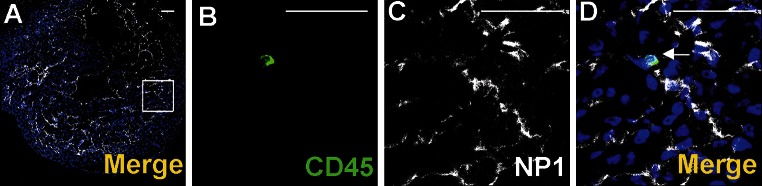
Location of CD45^+^/Np1^+^ cells close to the endothelium lining cardiac trabeculae. Confocal microscope images of sections from a 13 dpc heart stained with anti-CD45 (*green*) (**b**), anti-NP1 (*white*) (**c**) antibodies; merged panels present Hoechst staining of nuclei (*blue*) (**a**, **d**). The area marked with the *white box* (**a**) is enlarged in **d**. A CD45/NP1 double-positive cell (marked with the *white arrow*) is located among trabecular endothelial cells (**d**). *Scale bars* 50 µm

The endocardial endothelium expressed typical endothelial cell markers, such as CD31, Flk1, NP1, Fli1 and Tie2. In focal areas adjacent to the endocardial endothelium, numerous CD41^+^ cells were observed (Fig. 11a–j). These cells were mostly clustered close to endocardial cushions and were occasionally found in endocardial trabeculae (Fig. 7e–h). Cells expressing CD41/CD31/Fli1 were found occasionally in the atrial endothelium of mid-gestation (12 dpc) hearts (Fig. 11k–o). In addition, individual CD45^+^/NP1^+^ cells were localized in the endothelium of trabeculae (Fig. 12).

**Fig. 13:**
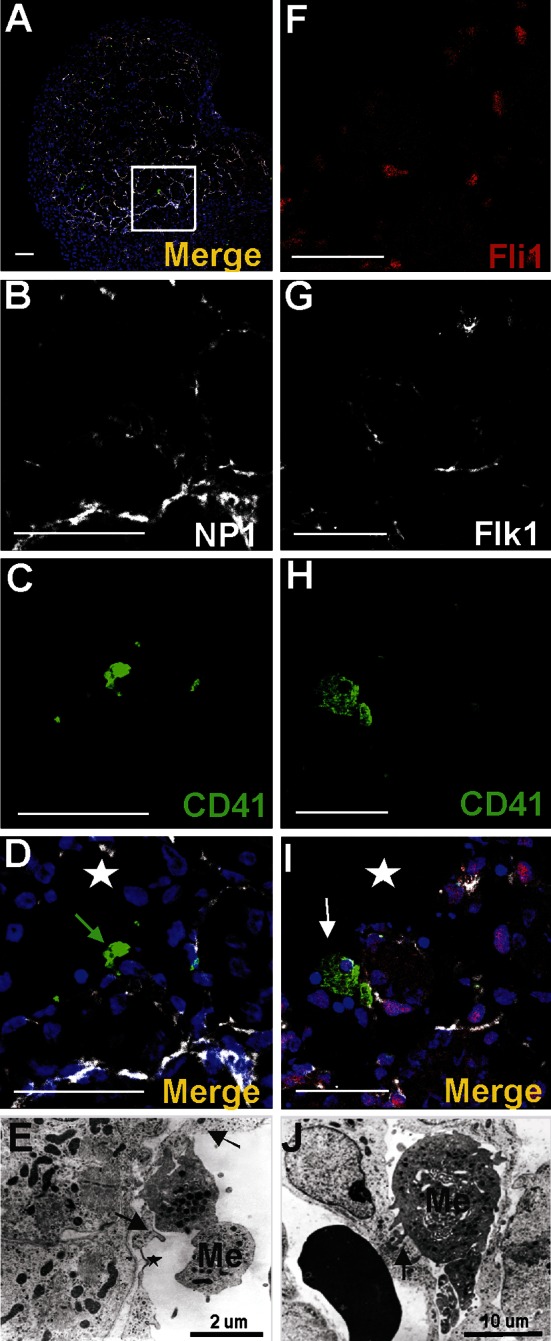
Megakaryocytes are attached to the endocardial endothelium within the lumen of a ventricle. Confocal microscopic panels (**a**–**d**, **f**–**i**) demonstrating sections of 13 dpc hearts stained with anti-CD41 (*green*) (**c**, **h**), anti-NP1 (**b**) or anti-Flk1 (**g**) (*white*) and anti-Fli1 (*red*) (**f**) antibodies; merged images that include Hoechst staining of nuclei (*blue*) are presented in **a**, **d**, **i**. The area *boxed* in **a** is enlarged in **d**. A CD41^+^ cell (*green arrow*) (**d**) and a CD41^+^ cell co-expressing the Fli1 transcription factor (*white arrow*) (**i**) adhere to the endocardial endothelium from the side of a cardiac cavity. *White stars* point to the lumen of a ventricle (**d**, **i**). Electron microscopy images (**e**, **j**) demonstrating the adherence of megakaryocytes and/or platelets (Me) to the endocardial endothelium (*black arrows*); prominent cytoplasmic protrusions toward the endothelium suggest penetration of a megakaryocyte to the subendocardium (**e**); the *black star* indicates a marked thinning of endothelial cell cytoplasm (**e**). *Scale bars* (panels **a**–**d**, **f**–**i**) 50 µm

Numerous large CD41-positive cells were found attached to the endocardial endothelium from the side of cardiac cavities. Some of them co-expressed Fli1 transcriptional factor (Fig. 13a–i).

Ultrastructural evaluation of endothelial lining of the endocardium showed that some endocardial fragments covering trabeculae were represented only by two plasma membranes (Fig. 13e) in which fenestrations were observed occasionally. Some platelets migrated to the myocardium via these fenestrations (Fig. 13e, j). Megakaryocytes with their protrusions were anchored to the endocardial endothelial cells and seemed to release platelets toward a cardiac cavity. Megakaryocytes found in the lumen of cardiac cavities were usually devoid of proplatelets.

### Clusters of cells expressing putative vasculogenic and hematopoietic progenitor markers emerge from the endothelium of atria and the outflow tract

**Fig. 14:**
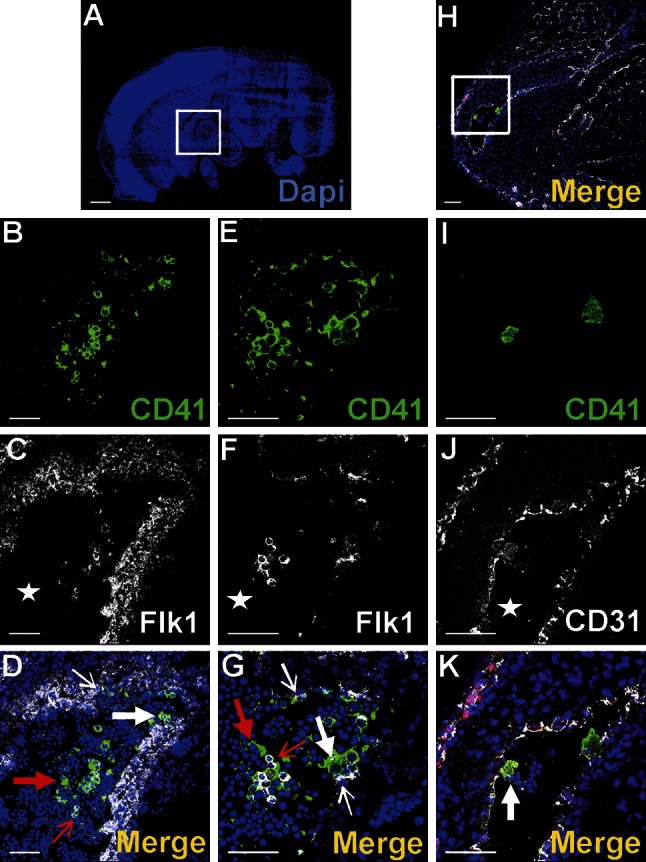
Clusters of cells expressing hematopoietic progenitor markers emerge from the endothelium of the outflow tract. Confocal microscope images of a 11.5 dpc (**a**–**g**) and a 13 dpc (**h**–**k**) heart sections. Cells are stained with anti-CD41 (*green*) (**b**, **e**, **i**) and anti-Flk1 (**c**, **f**) or anti-CD31 (*white*) (**j**) antibodies. **d**, **g**, **k** Merge panels include Hoechst staining of cell nuclei. The area located in the *white box* of panel **a** is enlarged on panels **d**, **g**, while the area framed in the *white box* of panel **h** is enlarged on panel **k**. *White stars* point to the lumen of an atrium (**c**, **f**) and to the outlow tract lumen (**j**). Red-stained cells in picture **k** are Fli1-positive. Double-positive CD41/Flk1 (**d**, **g**) or CD41/CD31 (**k**) cells adhere to the endothelium as indicated by thick *white arrows* (**d**, **g**, **k**). Some double-positive cells seem to originate from endothelium (*thin white arrows*) (**d**, **g**). Free-floating double-positive cells (*thin red arrows*) in the lumen of the atrium (**d**, **g**) and CD41-positive cells (*red arrows*) (**d**, **g**) are also visible*. Scale bar* (panel **a**) 500 µm, *Scale bars* (panels **b**–**h**) 50 µm

Cells expressing CD41 and Flk1 antigens were found in the atrial lumen and adjacent to atrial endothelium of 11.5 dpc hearts (Fig. 14a–g). These cells were located in the close vicinity to the endothelium, i.e., adhering to it, or were dispersed between endocardial endothelial cells. During this developmental stage, free-floating double-positive cells (CD41/Flk1), as well as cells possessing only CD41 antigen, were also visible in the lumen of atria.

Cells co-expressing CD41 and CD31 antigens were found also adhering to the outflow tract endothelium of 13 dpc hearts (Fig. 14h–k).

### Contrary to the fetal blood, fetal hearts are a source of the cells bearing putative vasculogenic and hematopoietic progenitor markers

**Fig. 15:**
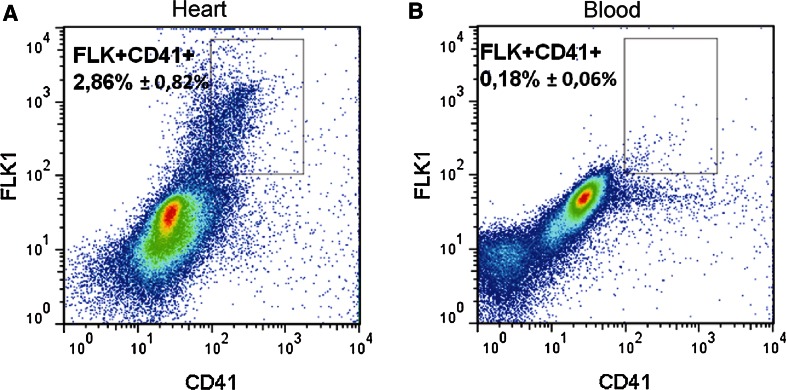
The cells bearing putative vasculogenic and hematopoietic progenitor markers are numerous in 12.5 dpc hearts. Flow cytometric analysis of CD41 and Flk1 double-positive cells. Cells isolated from hearts (**a**) or blood samples (**b**) were stained with anti-CD41-PE and anti-Flk1-APC antibodies. The rectangular gate marks a population of CD41/Flk1 double-positive cells isolated from hearts (**a**). This population is not present in blood samples (**b**). The mean population frequency obtained from six experiments is shown ± standard deviation

A population of double-positive (CD41/Flk1) cells has been detected in cells isolated from the heart. The frequency of this population was 2.68 ± 0.82 % in the analyzed samples. Only 0.18 ± 0.06 % of double-positive cells were detected in blood samples by applying the same analysis gates as for the heart samples, which probably represented instrument background. The population of double-positive cells was significantly (*p* < 0.01) more abundant in the heart samples in comparison with that in blood samples (Fig. 15a, b).

## Discussion

The most important finding of our study was to demonstrate that the embryonic mouse heart during midgestation (i.e., at the stages between 11.5 and 14 dpc) contains cells bearing hematopoietic progenitor markers such as: GATA2^+^/CD41^+^, GATA2^−^/CD41^+^, GATA2^+^/CD71^−^, GATA2^−^/CD71^+^, Fli1^+^/CD71^+^, Fli1^−^/CD71^+^, CD45^+^/Fli1^+^, as well as cells with phenotypes: CD41^+^/Flk1^+^, Flk1^+^/Fli1^+^, that we believe to possess also vasculogenic potential.

These cells were found scattered or forming clusters in a close association with the endothelium lining the endocardial cushions as well as cardiac chamber cavities (both atria and ventricles). Cells exhibiting similar characteristics (based on their phenotypes and morphology) were also found in the subepicardium, usually in scattered pattern outside of blood islands. We demonstrated, using a FACS analysis, that CD41/Flk1 double-positive cells of putative vasculogenic and hematopoietic potential comprise surprisingly high proportion (2.68 %) of entire heart cell population at 12.5 dpc. Whether these cells can really differentiate into vascular or/and hematopoietic cells needs further studies with ex vivo assay application.

Literature reports indicate that cells exhibiting CD31^+^/CD41^+^ or CD41^+^/Flk1^+^ phenotypes can be considered as having hematopoietic potential (Hirschi 2012; Nakano et al. 2013). Our observations thus suggest that putative hematopoietic progenitors are generated in situ in the endothelial lining of atria on 11.5 dpc, in the endocardial cushions on 12 dpc and in endocardial trabeculae on 13 dpc. In addition, cells of CD41/CD31/Fli1 triple-positive phenotype, with presumably hemato-vascular potential, were found to be dispersed in the atrial endothelium.

Nakano et al. (2013) have reported that CD31^+^/CD41^+^ cells, a subset of endocardial endothelial cells in the outflow cushion and the atria, have hemogenic potential earlier than in the aorta-gonad-mesonephros region, i.e., at embryonic days between 8.25 and 10.5. Therefore, we postulate, for the first time, that hemogenic potential described by Nakano et al. (2013) lasts longer than until day 10.5, at least until 11.5 dpc.

Transcription factor Fli1 has been reported to be expressed at a high level in the nuclei of the endothelial cells bearing both the CD31 and CD41 antigens as compared with other endothelial cells. This high Fli1 expression may indicate hemogenic activity of endothelial cells. Thus, we believe that it may serve as an additional marker for hemogenic endothelium identification. It is known that murine Fli1 is essential for embryonic development and that this transcription factor is detected in early precursors of hematopoietic and endothelial lineages (Maroulakou and Bowe 2000).

Double-positive cells of CD45/NP1 phenotype were located in a close vicinity to CD41^+^/Flk1^+^ cells of endocardial trabeculae of mouse hearts. Yamada et al. (2003) found that cells co-expressing these markers may be engaged in inducing angiogenesis. There is no information, whether they may also have any influence on the development of the hemogenic endothelium and on hematopoiesis.

Our results demonstrate that Flk1/CD41 double-positive scattered cells are present in the subepicardium of the 13-dpc heart. The subepicardial space is a particular region of the fetal heart where differentiation of various subsets of progenitor cells occurs (Wessels and Pérez-Pomares 2004). These cells appear during heart development and result from epithelial-mesenchymal transformation (EMT) of the epicardium. A subset of epicardial-derived cells differentiate in various types of different cells, including coronary endothelium, smooth muscle cells, interstitial fibroblasts and atrioventricular cushion mesenchymal cells (Wessels and Pérez-Pomares 2004; Maya-Ramos et al. 2013; Pires-Gomes and Pérez -Pomares 2013). Our purely morphological and immunohistochemical approach does not indicate what the source of cells expressing Flk1/CD41 phenotype is: whether they come from the epicardium-derived cells via the process of EMT or from extracardiac cells that invade the developing heart. Cells expressing Flk1/CD41 phenotype were described previously by other authors as a population of hemogenic endothelium, being the normal component of embryonic blood vessels (Mikkola et al. 2003; Nakano et al. 2013). Since we observed these cells in the subepicardium of 13 dpc mouse hearts, we can hypothesize for the first time on the existence of hematopoietic potential in the subepicardial cells. Neither the origin nor the fate of these cells is known and needs further studies.

Cells expressing a strong signal for the Gata2 transcription factor were found in this study in the subepicardium of prenatal hearts. These cells did not express other hematopoietic markers, excluding a few cells co-expressing CD71. Double-positive Gata2/CD71 cells presumably represent early erythroid precursors (Ferreira et al. 2005). The significance of the presence of Gata2^+^ cells in the subepicardium is unclear. It must be remembered that transcription factor Gata2 is required for the proliferation and survival of immature hematopoietic progenitors (Pimanda et al. 2007). Similarly to other genes involved in hematopoiesis, such as Fli1, GATA2 also participates in vascular development and is expressed in the endothelial cell line (Pimanda et al. 2007). We may speculate that Gata2-positive cells located in the subepicardium are early precursors of hematopoietic and/or endothelial lineages.

Cells expressing Flk1/Fli1 antigens located in the subepicardium might be also progenitors of angioblasts taking part in vasculogenesis. If these cells co-express also the Tie2 antigen, they are visible in the network of endothelial cells forming blood vessels in the region of interventricular sulcus. As reported earlier (Sato et al. 1995), Tie2 is an angiopoietin receptor expressed in developing vascular endothelial cells and particularly important for vascular network formation.

Blood islands located in the subepicardium of murine and human embryonic hearts (Hirakow 1983) have been considered to be important structural components of hematopoiesis and vasculogenesis prior to the establishment of a connection between the coronary and the systemic circulation. Their origin and fate is still unclear. It has been postulated that blood island formation results from in situ differentiation of progenitor cells. Ratajska et al. (2006) suggested that blood islands of the embryonic heart arise by aggregation of migrating nucleated red blood cells with migrating endothelial cells. Recently, Red-Horse et al. (2010) demonstrated that a population of endocardial cells may bud through the myocardium, pinch off and form endothelial spheres with entrapped blood cells, forming blood islands. We studied serial sections of multiple heart specimens, and we did not find any morphological signs which may confirm the hypothesis by Red-Horse and coauthors.

In this paper, we presented the first quantitative data on the number of blood islands at various stages of mouse heart development. We noticed that blood islands appeared first on the dorsal surface of the heart and that during later stages, their number was higher on the ventral surface of the heart. We found that blood islands are still present on the ventral surface of 13.5 and 14 dpc hearts, therefore, at the stages when coronary circulation is patent with the systemic circulation. The absence of blood islands on the dorsal surface of embryonic hearts at 13.5–14 dpc stages might be related to an integration of blood islands with blood vessels, as was observed in 12.5-dpc hearts (Fig. 2k, l, o, p). Various possible fates of blood islands are predicted by different investigators. First, blood islands are supposed to elongate into vascular tubes, which fuse, branch and coalesce to form vessels (Tomanek 2005). Second, as suggested by Hirakow (1983), blood islands are canalized and communicate with venous sprouts (probably with the middle cardiac vein of the human heart). Our strict morphological data, based on observations of semithin Epon-embedded sections stained with toluidine blue as well as confocal microscope analysis, seem to confirm their elongation and coalescence with angiogenic sprouts.

We also documented, to our best knowledge, for the first time that megakaryocytes are present within the lumen of cardiac blood islands. In earlier papers, proplatelets/platelets residing in blood islands were illustrated (Ratajska et al. 2009), which have been confirmed by Red-Horse et al. (2010). However, the site of proplatelet/platelet formation has not been documented. Megakaryocytes found inside of blood islands demonstrated different stages of differentiation, including very young as well as mature forms, i.e., the ones that release proplatelets and exhibit almost completely denuded cytoplasm.

It has been reported previously (Ratajska et al. 2009) that erythroblasts at different stages of differentiation reside inside blood islands. Our present work confirms this finding. Both hematopoietic lineages, erythroblastic and megakaryocytic found in the lumen of blood islands presumably, emerge from the same precursor cell, i.e., a megakaryoblastic/erythroblastic precursor (MEP) (Tober et al. 2007). The CD41 antigen, found in numerous cells located in the cardiac blood islands, is considered to be a marker of early differentiation steps of hematopoietic cells, including erythroblastic and megakaryoblastic pathways (Ferkowicz et al. 2003; Mikkola et al. 2003). Cells located in blood islands expressed also the Ter119 and CD71 markers. The former is specific for early proerythroblast to mature erythrocyte stages (Fraser et al. 2007), and the latter marks early erythroid precursors, the intermediate normoblast and reticulocytes (Fraser et al. 2007). Some cells possessing CD41 or CD71 antigens co-express transcription factors Fli1 or Gata2. The last one is expressed in broad spectrum of hematopoietic cells, with particular abundance in early progenitors, as well as in megakaryocytes (Kitajima et al. 2002). Our immunohistochemical studies with the use of various combinations of the antibodies presented above confirm that cells located in the lumen of blood islands represent various stages of hematopoietic development. Taking into consideration that mesenchymal-like cells, erythroblastic and megakaryoblastic lineages which are found in blood islands are at various levels of differentiation, blood islands might be sites of hematopoiesis.

However, based on strictly phenotypic characteristics, we postulate that the endothelium of cardiac blood islands does not possess hemogenic features, since in our present investigations, we did not find co-expression of endothelial (CD31, Flk1 or NP1) and hematopoietic (CD41) markers in blood island endothelium.

Based on our results, we can consider the developing heart as an organ of intensive migration of megakaryocytic and erythroblastic cells. We define morphologic sings of megakaryocyte migration from blood islands to the subepicardium and myocardium and from heart chambers to the subendocardial space (toward cardiomyocytes). Megakaryocyte migration through the endothelial lining of the endocardium may by facilitated by close apposition of endothelial cell plasma membranes equipped with fenestrations. Earlier electron microscopic studies show that megakaryocytes may pass through transendothelial apertures of 6 µm in diameter (Zucker-Franklin and Philipp 2000). Megakaryocytic ability to migrate in embryonic tissues has been broadly documented. Megakaryocytes were observed in embryonic peripheral blood before liver hematopoiesis is established as well as in mouse embryo at 10 and 11 dpc, in the lumen of vitelline vessels and in the visceral yolk sac (Matsumura and Sasaki 1988). Megakaryocytes were also found in other intraembryonic vessels and in chambers of the developing heart (Tober et al. 2007).

In our study, we observed surprisingly abundant megakaryocytes and platelets located in the lumen of blood islands but also in the developing cardiac blood vessels. Some CD41-positive megakaryocytes were anchored to the endocardial trabeculae. In these megakaryocytes, the demarcation membranes were visible under electron microscope, suggesting that proplatelet production may occur. Thus, in our study, endothelial cells of the prenatal heart may play a role as an anchoring apparatus for megakaryocytes that release platelets to the cardiac cavities. It has been reported earlier that platelets are also produced in the capillary bed of adult mouse lungs (Zucker-Franklin and Philipp 2000). Lungs represent the area of the first capillary bed encountered by cells leaving the bone marrow. Platelet production in lungs might be stimulated by phlebotomy or by administration of the thrombopoietin.

The function of platelets in the developing heart is unknown. Some reports imply that platelets regulate new blood vessel growth through the release of numerous pro- and anti-angiogenic growth factors including VEGF-A, thrombospondin and other mediators (Battinelli et al. 2011). Platelets may play the same role in the prenatal heart.

Our study also presents erythroblasts penetrating between endothelial cells of the blood islands indicating their migration; they may be subsequently positioned in the subepicardium in the close vicinity to blood islands. Erythroblasts have been previously found in the subepicardial space without any contact with the endothelium (Ratajska et al. 2009). This study confirms the same finding. Our study documented extrusion of the nuclei from some erythroblasts occurring in the subepicardial space. Free nuclei as well as fragments of cell cytoplasm were phagocytized, presumably by macrophages. This work demonstrates an abundance of cells expressing immunohistochemical and ultrastructural features of macrophages. Macrophages (CD68^+^/Lyve1^+^ scattered cells) were found in the subepicardium and in the myocardium of the prenatal hearts. Their presence in the developing heart is not only related to the removal of cellular debris, but might also be involved in angiogenesis (Wynn et al. 2013), as well as in lymphatic vessel development (Flaht et al. 2012).

We believe that this study can form a platform for future research on mechanisms governing processes of vasculogenesis and hematopoiesis especially with the use of transgenic animals as well as for developing and elaborating techniques that allow for the isolation of these progenitor cells from solid tissues for in vitro studies.

## References

[CR1] Antas VI, Al-Dress MA, Prudence AJ, Sugiyama D, Fraser ST (2013). Hemogenic endothelium: a vessel for blood production. Int J Biochem Cell Biol.

[CR2] Battinelli EM, Markens BA, Italiano JEJR (2011). Release of angiogenesis regulatory proteins from platelet alpha granules: modulation of physiologic and pathologic angiogenesis. Blood.

[CR3] Boisset JC, Van Capppellen W, Andrieu-Soler C, Galjart N, Dzierżak E, Robin C (2010). In vivo imaging of haematopoietic cells emerging from the mouse aortic endothelium. Nature.

[CR4] Drake CJ, Fleming PA (2000). Vasculogenesis in the day 6.5–9.5 mouse embryo. Blood.

[CR5] Ferkowicz MJ, Starr M, Xie X, Li W, Johnson SA, Shelley WC, Morrison PR, Yoder MC (2003). CD41 expression defines the onset of primitive and definitive hematopoiesis in the murine embryo. Development.

[CR6] Ferreira R, Ohneda K, Yamamoto M, Philipsen S (2005). GATA1 function, a paradigm for transcription factors in hematopoiesis. Mol Cell Biol.

[CR7] Flaht A, Jankowska-Steifer E, Radomska DM, Madej M, Gula G, Kujawa M, Ratajska A (2012). Cellular phenotypes and spatio-temporal patterns of lymphatic vessel development in embryonic mouse hearts. Dev Dyn.

[CR8] Fraser ST, Isern J, Baron MH (2007). Maturation and enucleation of primitive erythroblasts during mouse embryogenesis is accompanied by changes in cell-surface antigen expression. Blood.

[CR9] Hirakow R (1983). Development of the cardiac blood vessels in staged human embryos. Acta Anat.

[CR10] Hirschi KK (2012). Hemogenic endothelium during development and beyond. Blood.

[CR11] Kitajima K, Masuhara M, Era T, Enver T, Nakano T (2002). GATA-2 and GATA-2/ER display opposing activities in the development and differentiation of blood progenitors. EMBO J.

[CR12] Lancrin C, Sroczynska P, Stephenson C, Allen T, Kouskoff V, Lacaud G (2009). The haemangioblast generates haematopoietic cells through a haemogenic endothelium stage. Nature.

[CR13] Li W, Ferkowicz MJ, Johnson SA, Shelley WC, Yoder MC (2005). Endothelial cells in the early murine yolk sac give rise to CD41-expressing hematopoietic cells. Stem Cells Dev.

[CR14] Li Z, Lan Y, He W, Chen D, Wang J, Zhou F, Wang Y, Sun H, Chen X, Xu CH, Li S, Pang Y, Zhang G, Yang L, Zhu L, Fan M, Shang A, Ju Z, Luo L, Ding Y, Guo W, Yuan W, Yang X, Liu B (2012). Mouse embryonic head as a site for hematopoietic stem cell development. Cell Stem Cell.

[CR15] Maroulakou IG, Bowe DB (2000). Expression and function of Ets transcription factors in mammalian development: a regulatory network. Oncogene.

[CR16] Matsumura G, Sasaki K (1988). The ultrastructure of megakaryopoietic cells of the yolk sac and liver in mouse embryo. Anat Rec.

[CR17] Maya-Ramos L, Cleland J, Bressan M, Mikawa T (2013). Induction of the proepicardium. J Dev Biol.

[CR18] Mikkola HK, Klintman J, Yang H, Hock H, Schlaeger TM, Fujiwara Y, Orkin SH (2003). Haematopoietic stem cells retain long-term repopulating activity and multipotency in the absence of stem-cell leukaemia SCL/tal-1 gene. Nature.

[CR19] Mukouyama YS, James J, Nam J, Uchida Y (2012). Whole-mount confocal microscopy for vascular branching morphogenesis. Methods Mol Biol.

[CR20] Nakano H, Liu X, Arshi A, Nakashima Y, Handel B, Sasidharan R, Harman AW, Shin JH, Schwartz RJ, Conway SJ, Harvey RP, Pashmforoush M, Mikkole HKA, Nakano A (2013). Hemogenic endocardium contributes to transient definitive hematopoiesis. Nat Commun.

[CR21] Pimanda JE, Ottersbach K, Knezevic K, Kinston S, Chan WY, Wilson NK, Landry JR, Wood AD, Kolb-Kokocinski A, Green AR, Tannahill D, Lacaud G, Kouskoff V, Göttgens B (2007). Gata2, Fli1, and Scl form a recursively wired gene-regulatory circuit during early hematopoietic development. Proc Natl Acad Sci USA.

[CR22] Pires-Gomes AS, Pérez -Pomares JM (2013) The epicardium and coronary artery formation. J Dev Biol 186–202; doi: 10.3390/jdb1030186

[CR23] Ratajska A, Czarnowska E, Kołodzińska A, Kluzek W, Leśniak W (2006). Vasculogenesis of the embryonic heart: origin of blood island-like structures. Anat Rec A Discov Mol Cell Evol Biol.

[CR24] Ratajska A, Czarnowska E, Kołodzińska A, Jabłońska A, Stachurska E (2009). New morphological aspects of blood islands formation in the embryonic mouse hearts. Histochem Cell Biol.

[CR25] Red-Horse K, Ueno H, Weissman IL, Krasnow MA (2010). Coronary arteries form by developmental reprogramming of venous cells. Nature.

[CR26] Sato TN, Tozawa Y, Deutsch U, Wolburg-Buchholz K, Fujiwara Y, Gendron-Maguire M, Gridley T, Wolburg H, Risau W, Qin Y (1995). Distinct roles of the receptor tyrosine kinases Tie-1 and Tie-2 in blood vessel formation. Nature.

[CR27] Tober J, Koniski A, McGrath KE, Vemishetti R, Emerson R, Mesy-Bentley KKL, Palis J (2007). The megakaryocyte lineage originates from hemangioblast precursors and is a integral component both of primitive and of definitive hematopoiesis. Blood.

[CR28] Tomanek RJ (2005). Formation of coronary vasculature during development. Angiogenesis.

[CR29] Wessels A, Pérez-Pomares JM (2004). The epicardium and epicardially derived cells (EPDCs) as cardiac stem cells. Anat Rec A Discov Mol Cell Evol Biol.

[CR30] Wynn TA, Chawla A, Pollard JW (2013). Macrophage biology in development, homeostasis and disease. Nature.

[CR31] Yamada Y, Oike Y, Ogawa H, Ito Y, Fujisawa H, Suda T, Takakura N (2003). Neuropilin-1 on hematopoietic cells as a source of vascular development. Blood.

[CR32] Zucker-Franklin D, Philipp CS (2000). Platelet production in the pulmonary capillary bed: new ultrastructural evidence for an old concept. Am J Pathol.

